# Experimental Study and Finite Element Analysis on Load-Bearing Performance of Corrugated Steel–Concrete Composite Arch Structures

**DOI:** 10.3390/ma19204392

**Published:** 2026-10-19

**Authors:** Xiaoyu Xu, Hainan Fu, Xiong Zhou, Mengjie Hao, Yang Du

**Affiliations:** School of Engineering and Technology, China University of Geosciences (Beijing), Beijing 100083, China

**Keywords:** arched corrugated steel–concrete composite structure, loading test, synergistic effect, finite element analysis, structural equivalent calculation

## Abstract

**Highlights:**

- A new arch composite structure composed of corrugated steel and concrete is proposed, and the structural loading test is carried out.
- A three-dimensional finite element model of composite structure is established, which can accurately reflect the actual stress and failure evolution process of the structure.
- It is proposed that the composite structure is equivalent to a homogeneous new composite structure, which can accurately characterize the mechanical characteristics of the composite structure specimen under mid-span load.

**Abstract:**

This study compares the mechanical characteristics of an arched corrugated steel–concrete composite structure (CSC), a concrete-only structure (C), and a corrugated steel-only structure (CS) through loading tests. By integrating three-dimensional numerical simulation analysis, it systematically investigates the load-bearing performance, deformation characteristics, and failure mechanisms of the three structures. The test results indicate that the ultimate bearing capacity of CSC reached 216 kN, representing increases of 517% and 52% compared with C and CS, respectively. The overall flexural stiffness and ductility were significantly enhanced, and the ultimate bearing capacity exceeded the linear superposition of the individual capacities of C and CS, demonstrating a positive synergistic force. A three-dimensional finite element numerical model was established. Finite element analysis was conducted under boundary and loading conditions identical to those used in the loading tests. The results indicate that the relative error between the simulated and experimental ultimate bearing capacities did not exceed 2.3%. The load–displacement curves exhibited a variation trend basically consistent with the experimental results, demonstrating that the model can reflect the actual mechanical behavior and failure evolution process of the structure with reasonable accuracy. The damage distribution maps elucidate the three-stage damage evolution pattern of the composite structure: damage initiates at the corrugated steel–concrete contact interface, subsequently propagates upward through the concrete, and ultimately extends to the concrete surface, causing cracking and interfacial delamination and leading to structural failure. Finally, to simplify the finite element numerical analysis procedure and improve computational efficiency, three equivalent modeling schemes for the composite structure were proposed. Comparative analysis revealed that when the composite structure was equivalent to a homogeneous novel composite material structure, the deviation in ultimate bearing capacity between the equivalent structure and the test specimen was controlled within 16%, and the maximum displacement deviation was controlled within 1.35%, while the deformation characteristics remained in good agreement. The proposed equivalent method can provide a reference for equivalent modeling in the design and FE numerical analysis of similar composite structures.

## 1. Introduction

Corrugated steel plates are thin-walled structural members formed by cold roll forming, which creates continuous corrugations on the plate surface. The geometric restraint provided by these corrugations gives the member much higher out-of-plane stiffness and buckling resistance in the direction perpendicular to the corrugations than those of a flat plate. These plates also offer advantages such as light weight, high strength, and easy assembly and construction [1], and they have therefore been widely used in various engineering structures, including buried culverts, utility tunnels, and tunnel supports [2]. Existing studies have shown that corrugation shape (wave height, wave spacing, and wave width) and plate thickness are key parameters determining the mechanical performance of corrugated steel. Increasing the corrugation depth can significantly reduce the slenderness ratio of the structure, causing the failure mode to change from global buckling to elastoplastic local buckling [3]. Modal analysis further confirms that introducing corrugations can significantly improve the out-of-plane stiffness of members and thereby increase the natural frequency of the structure [4]. In addition, the use of high-strength steel and deep corrugated steel profiles can reduce the cross-sectional area by 30–40% while maintaining adequate local buckling performance [5]. In terms of structural form, transversely arranged corrugated steel plates can effectively suppress premature local buckling of members, enable the release of axial loads, and provide lateral confinement to the internally filled concrete [6,7]. Based on this confinement effect, stress–strain constitutive models for sandwiched concrete and calculation methods for the bearing capacity of short columns have been successively established [8,9]. It should be noted that the favorable interfacial bond performance between corrugated steel and concrete provides the basis for their composite action. Existing experimental studies have shown that the failure modes of the corrugated steel–concrete interface can be categorized into two types: bond–anchorage failure and steel plate yielding failure. The ratio of chemical adhesion, friction, and mechanical interlocking in the interfacial bearing capacity is approximately 1:0.017:0.22 [10]. Corrugated steel–concrete composite structures developed from the above research findings exhibit excellent load-bearing capacity, stiffness, and ductility [11,12], and have become a research hotspot in the field of composite structures.

Considerable research has been conducted on loading tests and numerical analyses of corrugated steel and corrugated steel–concrete composite structures. In terms of loading tests, axial compression tests have been widely applied to concrete-filled corrugated steel tubular columns [6], double-skin concrete-filled corrugated steel tubular columns [9,13], special-shaped columns connected by double corrugated steel plates [7], and various composite walls [14,15]. The effects of parameters such as slenderness ratio, hollow ratio, plate thickness, and material strength on the load-bearing capacity and ductility of these structures have been systematically investigated. Research on the flexural behavior of corrugated steel–concrete composite structures has focused extensively on structural forms such as corrugated steel-reinforced concrete pipes [16,17], corrugated steel–fabric-formed concrete composite supports [12,18,19], tunnel lining arches strengthened with corrugated steel [20], and corrugated steel–concrete composite bridge decks [21]. The shear performance and other properties of structures are often revealed by shear tests [22], low-cycle reversed loading tests [23,24], and hysteretic loading [25]. Dedicated studies have also examined the mechanical responses of composite structures under extreme service conditions, such as impact [26], contact explosion [27], and fatigue [28,29]. Full-scale in-situ tests have further verified the service performance of multi-span corrugated steel arch culverts [30], multi-arch corrugated steel utility tunnels [31], and tunnel linings in seasonally frozen regions [32] under actual conditions. In finite element numerical analysis, existing studies have generally established a systematic procedure. This procedure includes building three-dimensional finite element models, validating model accuracy against test results [33,34], and using numerical models to reveal the influence of material parameters and composite configurations on the mechanical performance of structures [11,35,36]. For different loading conditions such as axial compression, bending, and shear, calculation formulas for bearing capacity and stiffness have also been successively proposed [21,24]. Although existing studies are relatively extensive, three shortcomings remain. Firstly, existing studies have mostly focused on line-type or plate-type members such as columns, walls, beams, and slabs. Systematic loading tests on arched corrugated steel–concrete composite structures are still relatively scarce. Secondly, most finite element models have not analyzed the damage evolution process of corrugated steel–concrete composite structures. Moreover, the synergistic working mechanism between corrugated steel and concrete affects the failure modes, displacement ductility, and internal force redistribution of composite arches. However, this influence has not been thoroughly explained through mutually corroborated experimental and numerical studies.

To reduce the computational cost of refined finite element analysis and simplify engineering design procedures, equivalent theories have been widely applied in research on corrugated steel structures. In terms of geometric equivalence, the orthotropic model has been proven to be a reliable alternative to explicit modeling of corrugation geometry. This model can reduce computation time while ensuring the accuracy of predicted values [36]. The equivalent orthotropic plate method has also been used to establish a simplified three-dimensional refined model for seismic analysis of large-span corrugated steel utility tunnels. The calculated results agree well with the test results [37]. In terms of member system equivalence, corrugated steel plate shear walls can be equivalent to closely spaced multi-horizontal-bar or multi-vertical-bar models connected to frame beams and columns based on the shear mechanism. Through the stiffness alignment principle, the initial equivalent stiffness is made exactly consistent with the calculation results of the refined shell element model [38]. The equivalent truss model can achieve clear separation of internal forces between the wall panel and the frame. It can be used to derive a unified calculation formula for the shear capacity of shear walls under complex stress states [39,40]. In the fields of stiffness equivalence and constitutive equivalence, a fitted formula for equivalent flexural stiffness has been developed. It accounts for rotational restraint at beam ends and concrete cracking in negative moment regions, providing a feasible approach for the simplified elastic calculation of composite beam deflection [41]. A table of equivalent stiffness coefficients for corrugated steel plates and a two-stage rotational stiffness model for flange joints were established based on full-scale tests. These models provide support for developing a plane strain-spring simplified calculation method for prefabricated corrugated steel primary support structures [42]. An equivalent stress–strain model was proposed for concrete-filled steel tubular members [43]. A constitutive model was developed for sandwiched concrete [8]. These two models provide a theoretical basis for fiber element analysis of composite members. However, the existing equivalent methods are mainly developed for flat-plate shear walls, composite beams, or tunnel support structures. Most of them focus on stiffness equivalence in the elastic stage or on a single loading condition. Equivalent modeling research on arched corrugated steel–concrete composite structures remains scarce.

Compared with existing studies, the novelty of this paper is as follows. This paper focuses on the synergistic working behavior between corrugated steel and concrete, and analyzes its influence on the failure mechanism, displacement ductility, and ultimate bearing capacity of the composite arch. It aims to clarify the positive synergistic effect, namely that the ultimate bearing capacity of the composite structure exceeds the algebraic sum obtained by linear superposition of the ultimate bearing capacities of the individual structures. Based on the test results, a three-dimensional numerical model was established to clarify the damage evolution law of the composite structure. To simplify the numerical analysis procedure, a structural equivalence scheme was further proposed, and the accuracy of the model was verified. The research results can provide a reference for the design and engineering application of arched corrugated steel–concrete composite structures.

## 2. Materials and Methods

### 2.1. Specimen Structure and Material Properties

#### 2.1.1. Structural Section and Geometric Characteristics of the Specimen

**Figure 1:**
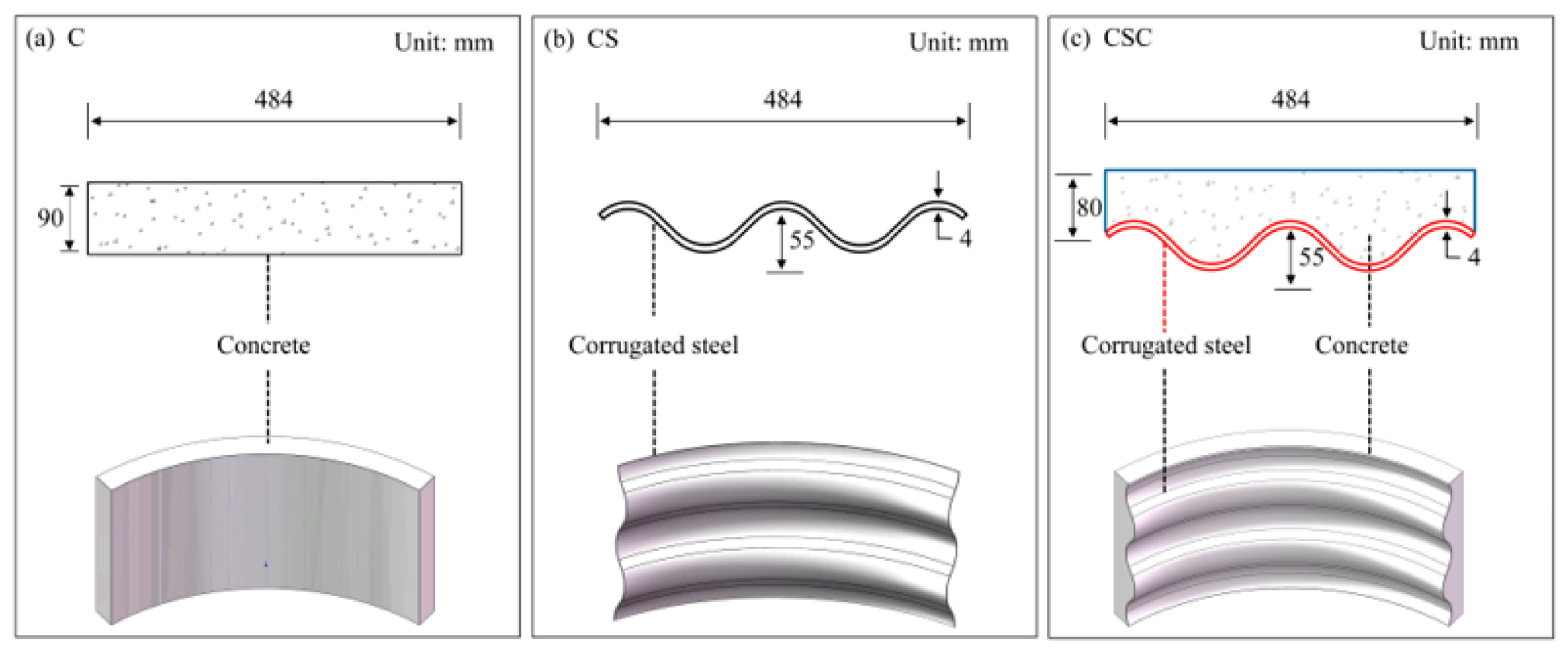
Structural section and geometric characteristics for (**a**) C, (**b**) CS, and (**c**) CSC.

The specimens in this study include a concrete specimen (C), a corrugated steel specimen (CS), and a corrugated steel–concrete composite specimen (CSC). Figure 1 shows the cross-sectional parameters of each structure. The corrugated steel in CS and CSC has identical geometric parameters. The corrugation wavelength, wave height and thickness are 200 mm, 55 mm, and 4 mm, respectively.

#### 2.1.2. Material Properties of Specimens

**Figure 2:**
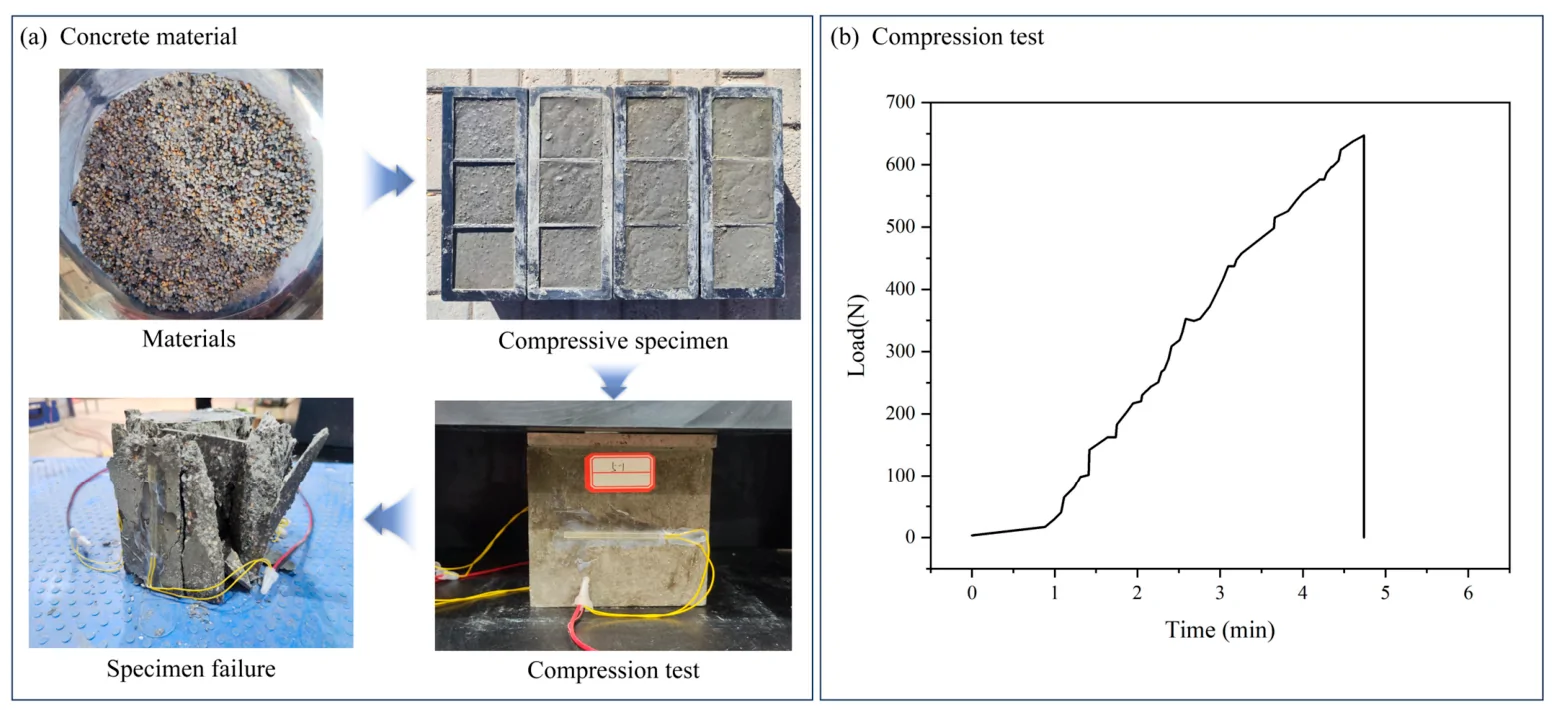
(**a**) Concrete material and (**b**) compression test.

The corrugated steel in specimens CS and CSC was made of Q235 carbon structural steel specified in GB/T 700-2006 [44]. Its mass density is 7850 kg/m^3^, its yield strength is 235 MPa, and its elastic modulus is 206 GPa. The steel structure materials and test specimens are from Hengshui Qijia Engineering Materials Co., Ltd., Hengshui, China. The concrete was prepared from mixed materials, and the mass percentages of its components are listed in Table 1. The concrete material comes from Guiyang Zhuonengda Special Building Materials Factory, Guiyang, China. To determine the compressive strength and elastic modulus of the concrete material, cube specimens were prepared and cured under standard curing conditions for 7 days before parameter measurement. According to the calculation methods specified in GB/T 50081-2019 [45] and GB 50010-2010 [46], the compressive strength and elastic modulus of the concrete were determined to be 56.2 MPa and 35,460 MPa, respectively. The concrete composition and the compressive test process are shown in Figure 2. It should be noted that steel tensile tests, concrete tensile tests, and interfacial bond characterization were not performed in this study. The strength of concrete obtained after 7 days of curing is not equivalent to the standard 28-day strength, and thus the absolute bearing capacity of the specimens may be underestimated. The relevant test data and conclusions obtained using the 7-day cured concrete strength in this study are mainly intended for comparison under identical conditions.

### 2.2. Loading Equipment and Scheme

**Figure 3:**
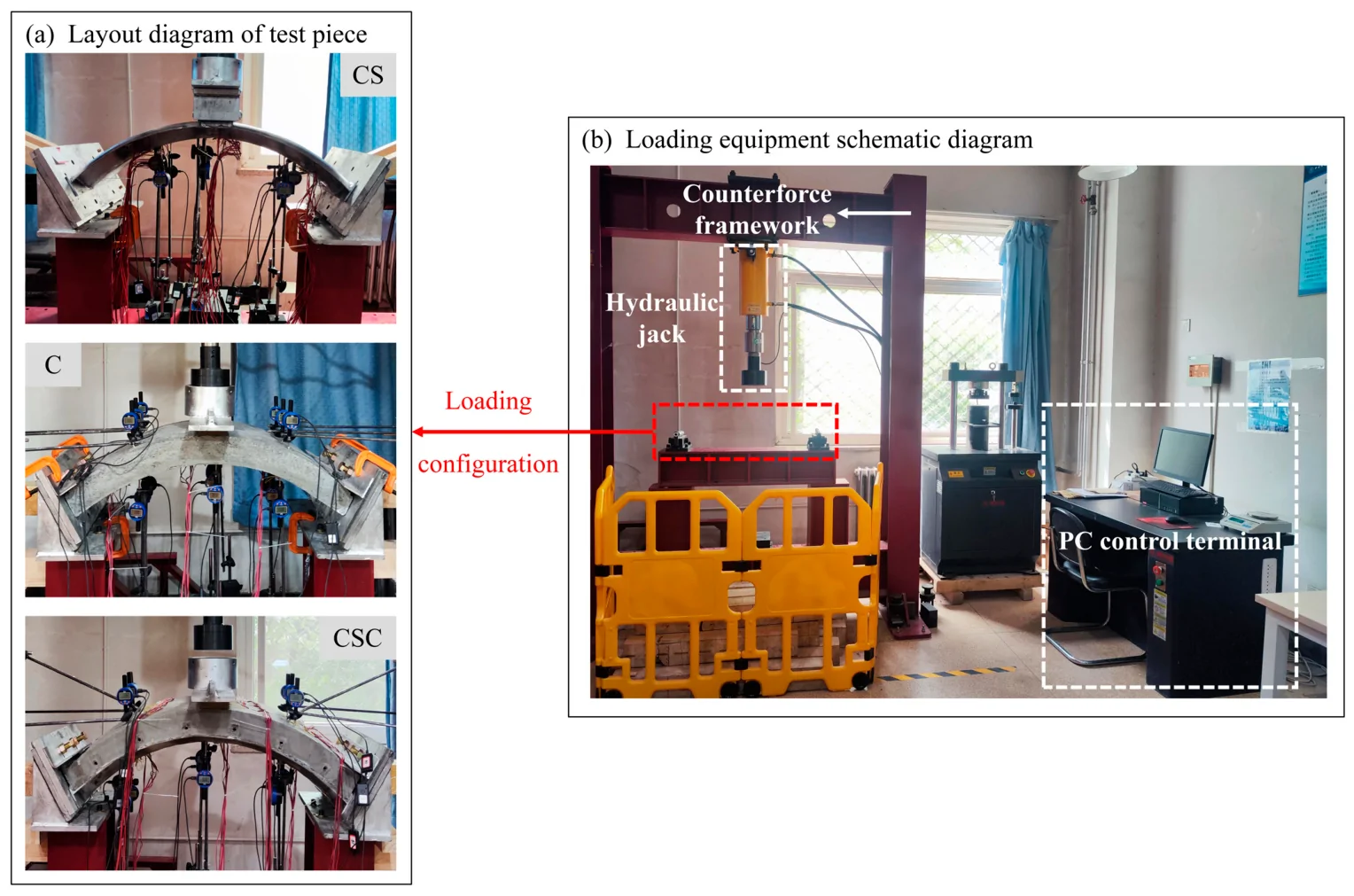
Loading test diagram (**a**) layout of the test piece and (**b**) loading equipment schematic diagram.

In this study, loading tests were conducted using a self-balancing reaction frame. This frame is from Jinan Hengle Xingke Instrument Co., Ltd., Jinan, China. The maximum applied load was 300 kN, and the maximum loading displacement was 300 mm. Figure 3 shows the loading test equipment and the loading arrangements of the three specimens. The test setup mainly consists of a reaction frame, hydraulic jacks, and fixed supports. It applies loads through a PC-controlled hydraulic pump driven by a servo motor, using a displacement-controlled loading mode. Monotonic loading was applied throughout the test without any unloading. Loading was terminated and the test ended when the applied force could no longer reach the peak load of the previous stage or when the structure failed.

For specimens C and CSC, flat rubber pads were placed between the jack and the specimen to transfer the load. For specimen CS, a rubber pad matching the corrugation profile of the corrugated steel was used at the jack–specimen interface. These different pad geometries were primarily intended to ensure uniform loading at the crown of the arched specimens.

### 2.3. Monitoring Scheme

**Figure 4:**
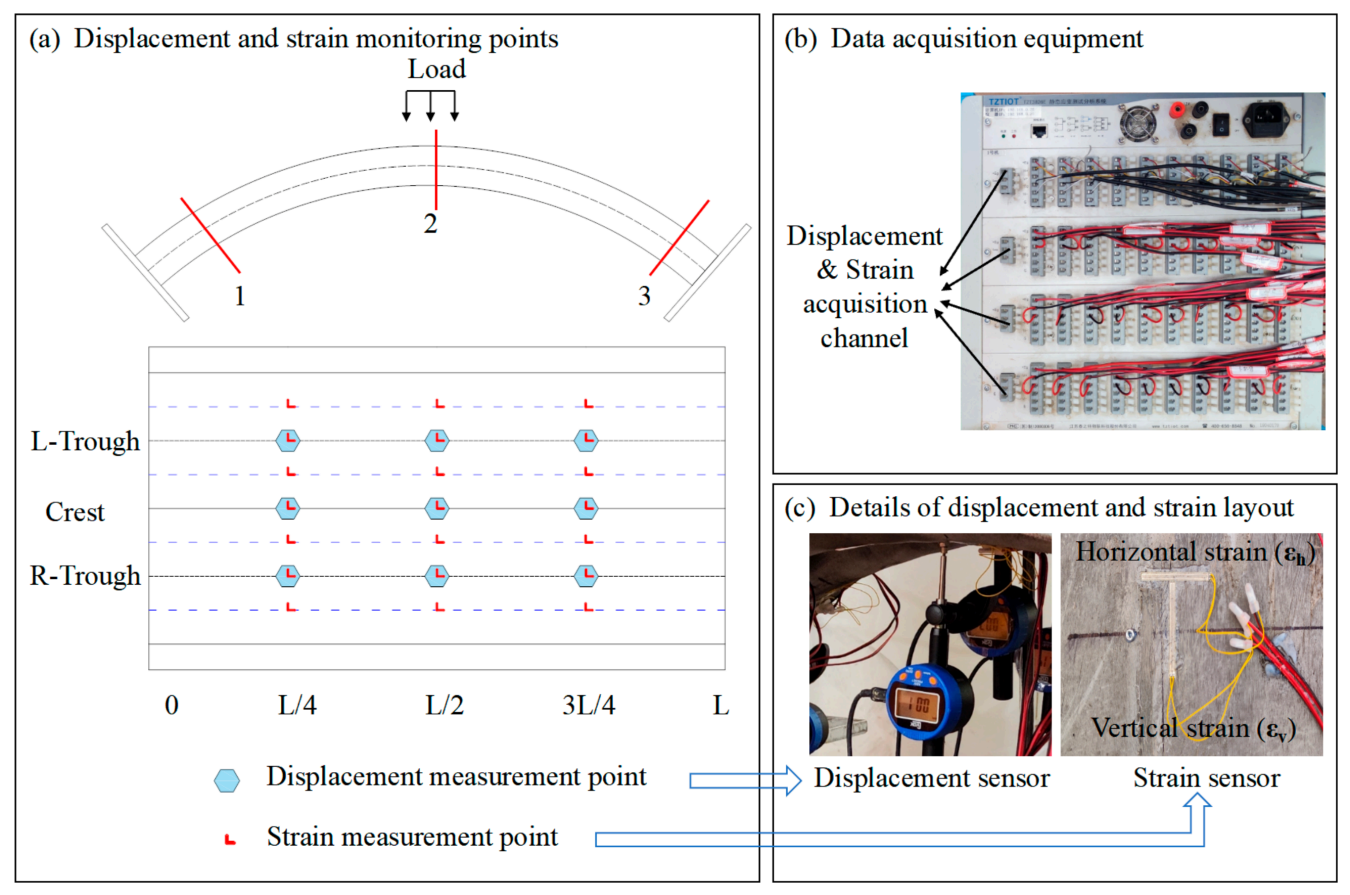
Specimen strain and displacement test point layout. (**a**) displacement and strain monitoring points and (**b**) data acquisition equipment and (**c**) details of displacement and strain layout.

The test measurements in this study included jack loading force, specimen displacement, and specimen strain. The jack loading force was automatically measured and recorded by a PC. Specimen strain data were acquired using electrical resistance strain gauges. Strain gauges of type 120-50AA were used for concrete strain measurement, and strain gauges of type 120-3AA were used for corrugated steel strain measurement. The strain and displacement sensor data were transmitted to a PC and stored via a TZT3826E data acquisition device. The manufacturer of the equipment is Jiangsu test electron equipment manufacturing Co., Ltd., which is located in Jingjiang, China. Figure 4 shows the locations of the strain and displacement measurement points. Three monitoring sections were selected, designated as Section 1 and Section 2, and 3 from left to right. Section 2 is located at the midpoint of the specimen, and Section 3 is symmetrically distributed with respect to Section 1. Taking specimen CS as an example, nine displacement measurement points and 21 strain measurement points were arranged on the specimen section. At each strain measurement point, mutually perpendicular circumferential and longitudinal strain gauges were installed. For CSC, the strain and displacement measurement points on the corrugated steel were arranged at the crest and trough positions. The concrete strain and displacement measurement points were located at positions corresponding to the crests and troughs of the corrugated steel in Section 1 and Section 2. The displacement and strain monitoring points in specimen C were arranged at exactly the same locations as those for the concrete in CSC.

## 3. Results

### 3.1. Structural Failure Characteristics

**Figure 5:**
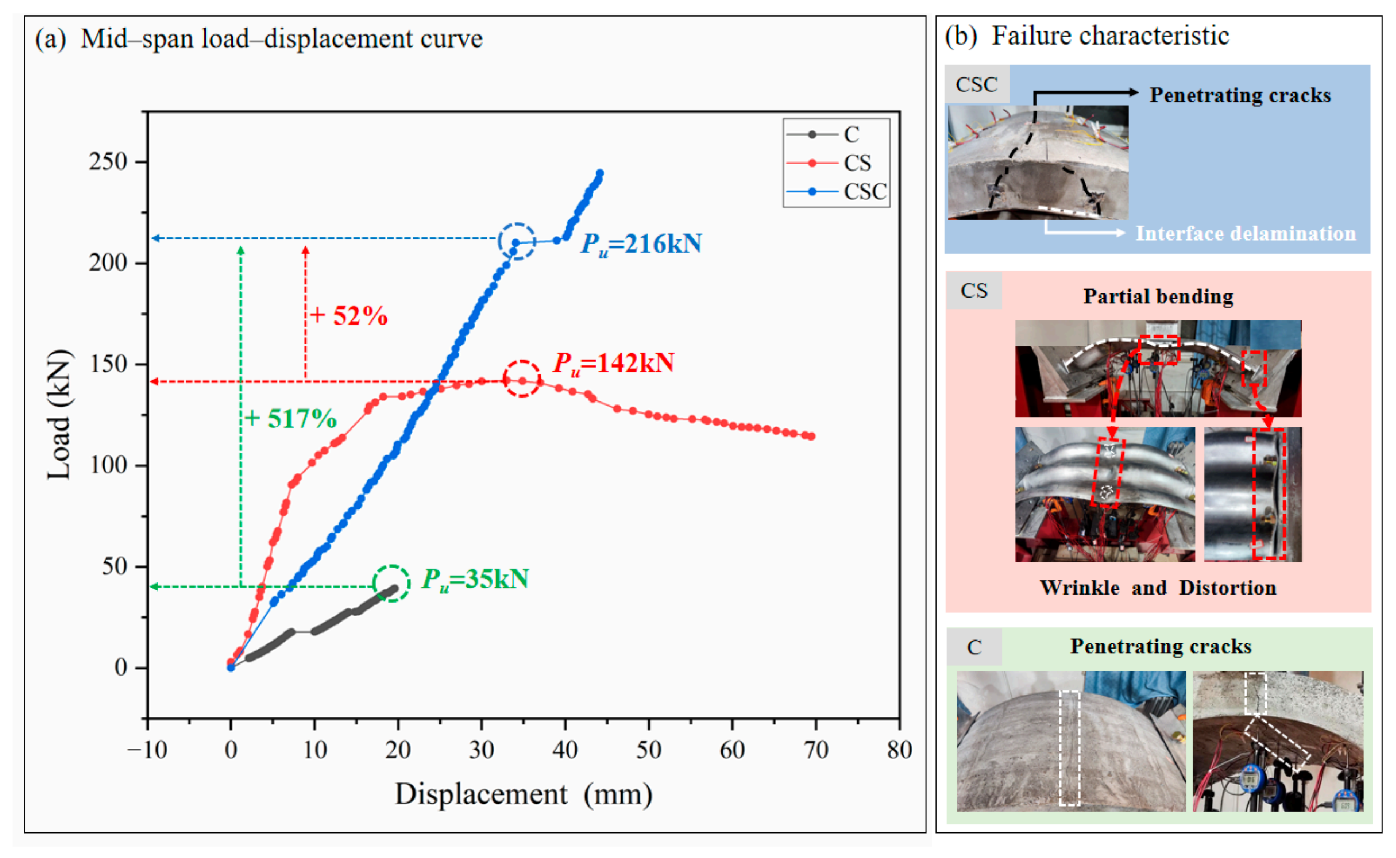
Mid-span load–displacement curve and failure characteristics of the structure. (**a**) mid-span load–displacement curve and (**b**) failure characteristic.

Figure 5a shows the mid-span load–displacement curves and failure characteristics of the three specimens during loading. The ultimate bearing capacities of specimens C, CS, and CSC were 35 kN, 142 kN, and 216 kN, respectively. Compared with C and CS, the ultimate bearing capacity of CSC increased by 517% and 52%, respectively. During the loading process of the test, all specimens successively exhibited three characteristic deformation stages: the elastic stage, the strengthening stage, and the yield–failure stage.

As the load increases, the mid-span displacement of the CSC specimen first increases rapidly, then remains stable, and finally continues to increase. This indicates that the structure has excellent initial stiffness and deformation capacity. A comparison of the load–displacement curves of the CS and CSC specimens shows that the CSC structure exhibited better ductility after reaching the ultimate load. This suggests that the composite action between concrete and corrugated steel enhances both the stiffness and bearing capacity of specimens C and CS. At the ultimate state, this enhancement effect on the mechanical performance of the composite structure becomes more pronounced.

Figure 5b shows the failure modes of the three specimens at ultimate load. After loading, all specimens underwent varying degrees of flexural deformation, with local failure occurring in different regions. For specimen C, a full-section through crack formed in the mid-span region upon reaching the ultimate load, and the specimen exhibited overall brittle failure. For specimen CS, significant local bending and plastic deformation developed in the mid-span region at the ultimate load. The loading surface showed obvious wrinkling, and the bolted connections exhibited clear deformation. Compared with specimens C and CS, specimen CSC exhibited higher flexural stiffness and maintained smaller deformation at higher load levels. Upon reaching the ultimate load, through cracks appeared in the concrete loading zone of CSC, crack propagation occurred along the concrete–corrugated steel interface, and concrete spalling was observed.

The test results show that all specimens underwent varying degrees of flexural deformation after loading. The deformation was particularly significant at the mid-span section and the bolted connections at the arch springing. The local failure modes were mainly manifested as buckling instability in the mid-span region. Specifically, edge tensile cracking occurred in specimen CS, while interfacial delamination between concrete and corrugated steel occurred in specimen CSC. Therefore, a reasonable selection of the composite configuration between corrugated steel and concrete can effectively enhance the overall mechanical performance and local damage resistance of the specimens.

### 3.2. Load and Structural Displacement

**Figure 6:**
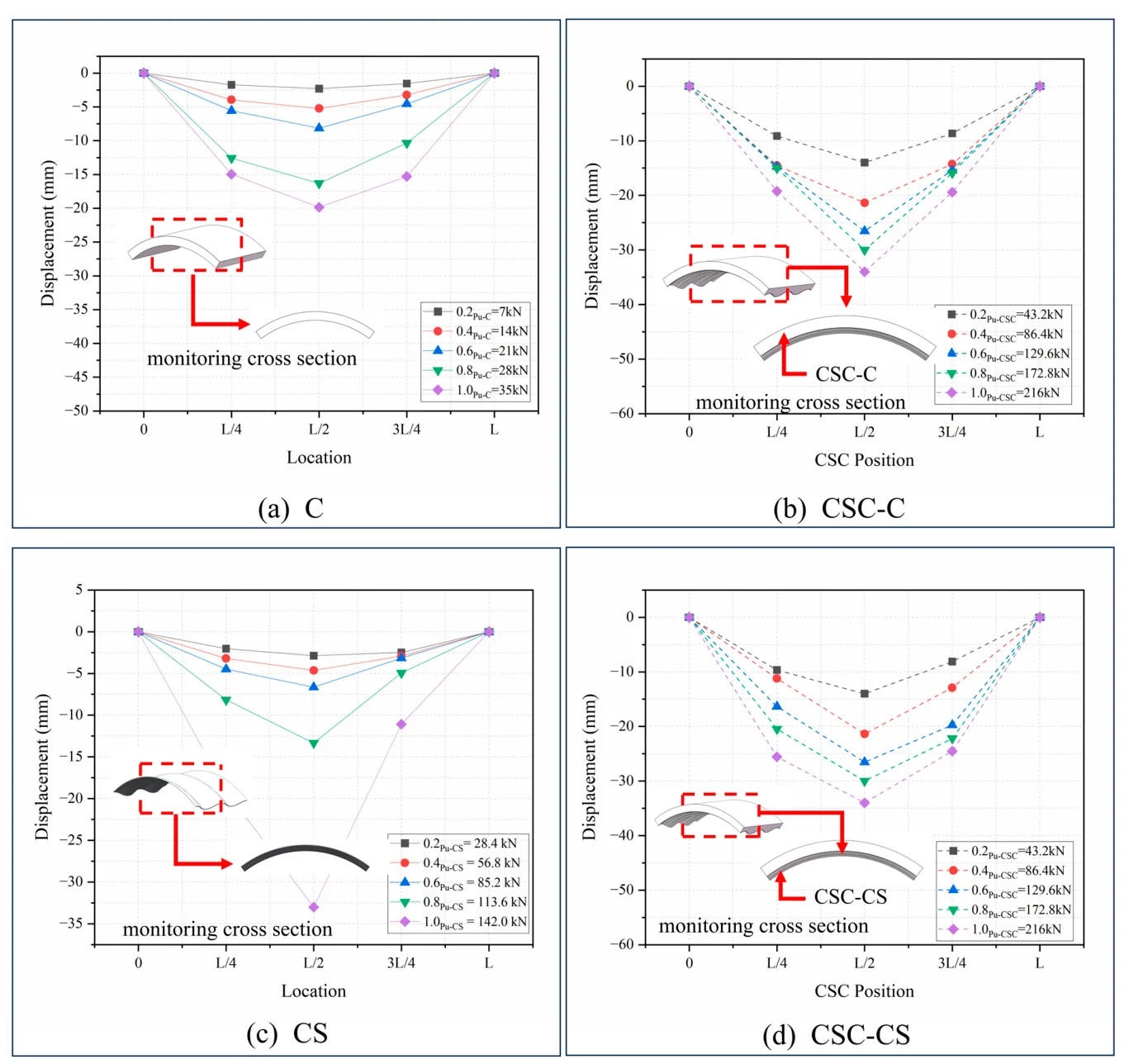
Structural displacement distribution under different loads. (**a**) C, (**b**) CSC-C, (**c**) CS, and (**d**) CSC-CS.

Figure 6 presents the displacement distributions along the span for the three arched specimens C, CS, and CSC under different load levels (0.2*P_u_*, 0.4*P_u_*, 0.6*P_u_*, 0.8*P_u_*, 1.0*P_u_*). All three specimens exhibit a deformation mode characterized by downward deflection at mid-span (Section 2) and upward bulging on both sides (Section 1 and Section 3), with the maximum vertical displacement occurring at mid-span (Section 2). The structures exhibited considerable differences in mechanical performance. As shown in Figure 6a, the ultimate bearing capacity of specimen C is *P_u_* = 35 kN, and the corresponding mid-span displacement is 20 mm. The concrete specimen, which had low flexural stiffness, exhibited a relatively short elastic stage during loading. It showed elastoplastic behavior at an earlier stage, and its strengthening stage was not obvious. After reaching the ultimate load, it rapidly entered the yield–failure stage, accompanied by significant crack propagation. As shown in Figure 6c, the ultimate bearing capacity of specimen CS is *P_u_* = 142 kN, and the corresponding mid-span displacement at ultimate load is 35 mm. Due to its relatively low flexural stiffness, the specimen entered the elastoplastic stage early in the loading process. Figure 6b,d show the load–displacement curves of the concrete and corrugated steel in the CSC specimen, respectively. The CSC specimen had the highest bearing capacity, reaching *P_u_* = 216 kN. As shown in Figure 6b, compared with specimen C, the vertical displacement of the concrete in the composite structure increased significantly with the inclusion of CS. As shown in Figure 6d, compared with specimen CS, the rate of change in the vertical displacement of the corrugated steel decreased significantly owing to the presence of the concrete. This indicates that the corrugated steel and concrete can form an effective synergistic load-bearing action. The concrete provides lateral confinement to the corrugated steel, inhibits the development of local buckling deformation, delays the onset of the plastic stage, and effectively reduces the deformation rate of the corrugated steel under the same load. In turn, the corrugated steel provides rigid support to the concrete, effectively restricts crack propagation, and enhances the deformation capacity of the concrete, enabling the composite structure to sustain higher load levels.

Combined with the failure characteristics described in Section 3.1, a high displacement growth rate of CSC is accompanied by concrete cracking and debonding of the corrugated steel–concrete interface. As shown in Figure 6, when the load exceeds 0.4*P_u_*, the local stiffness of the composite specimen begins to decrease. A comparison of the displacements of the three specimens at the same load level shows that the mid-span displacement of CSC is significantly smaller than those of C and CS. This indicates that the composite action between the two materials produces a positive synergistic effect, effectively improving the overall flexural stiffness and ductility of the structure. The mid-span displacement of the CSC specimen at ultimate load is larger than that of the two single-material structures, further confirming the superior ductility of the composite structure compared with the single-material structures.

### 3.3. Load and Structural Strain

**Figure 7:**
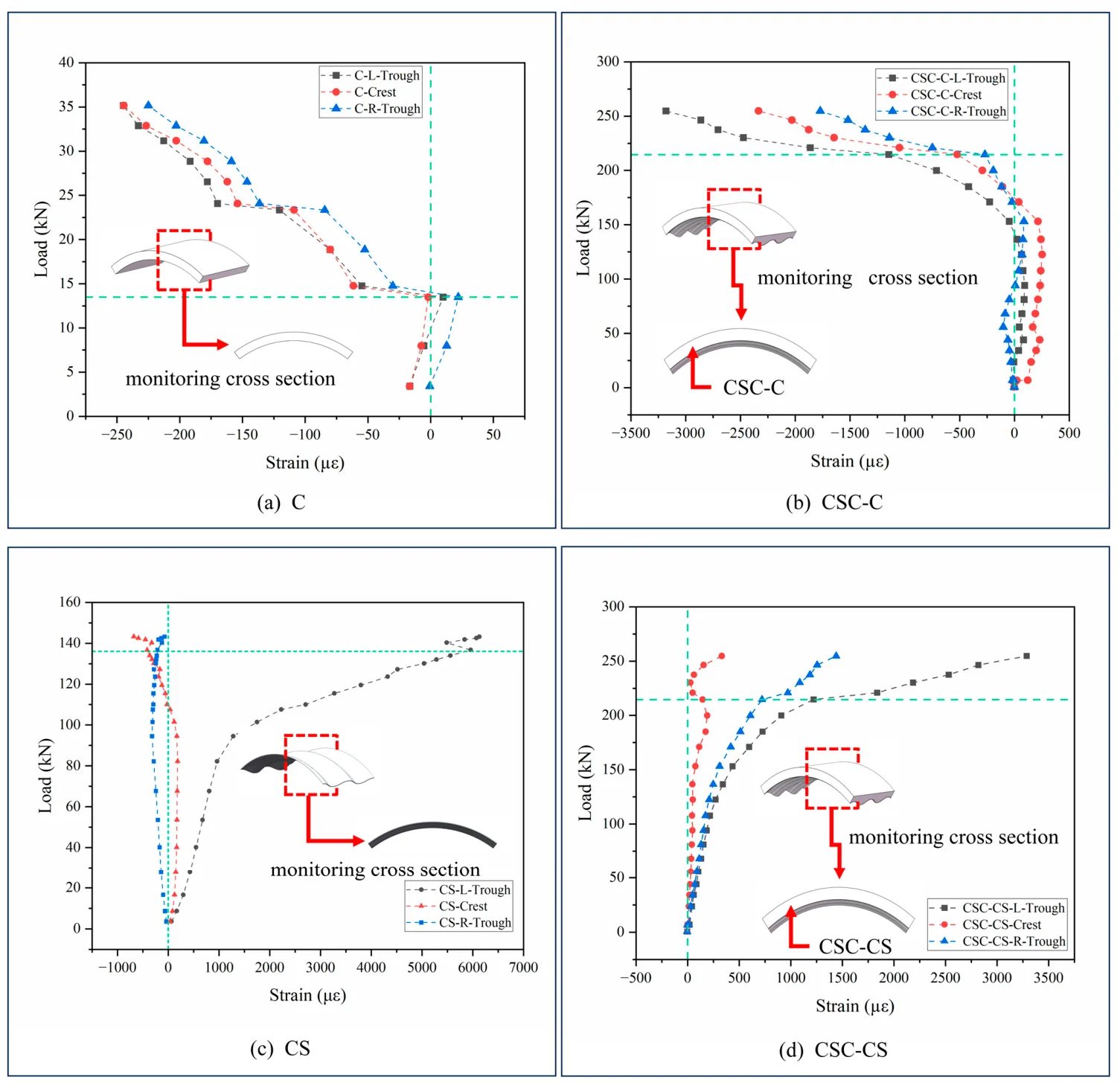
Transverse strain distribution of mid-span section of corrugated steel–concrete composite structure. (**a**) C, (**b**) CSC-C, (**c**) CS, and (**d**) CSC-CS.

Figure 7 presents the load–strain distributions at Section 2 of the three specimens during loading. The strain data in Figure 7 correspond to the strain data of the three monitoring points, Crest, L-Trough, and R-Trough, shown at Section 2 in Figure 4 throughout the loading process. The load points marked by green dashed lines in Figure 7 correspond to abrupt strain changes during loading, reflecting the failure characteristics of the structures. As shown in Figure 7, the strain values of all three structures gradually increase with increasing load. The strain is positive when the structure is in tension and negative when in compression. The strain values and their variation patterns differ significantly under different loads. As shown in Figure 7a, for the concrete-only specimen C, due to its relatively low flexural stiffness, an obvious abrupt change in strain occurs when the load reaches 0.6*P_u_*. This indicates that specimen C has cracked and rapidly entered the failure stage. As shown in Figure 7c, for the corrugated steel-only specimen CS, the strains at different positions of the corrugated steel differ significantly. The strain growth rate at the trough on the tensile side of the mid-span section is noticeably higher than that at the crest on the compressive side. When the load approaches the ultimate bearing capacity, the strain of the corrugated steel at the tensile side of the mid-span reaches the material yield strain first. Local plastic deformation then continues to develop, ultimately causing buckling instability in the mid-span region.

For specimen CSC, the strain distribution across the section fully reflects the synergistic load-bearing behavior of the two materials. The concrete mainly carries the compressive strain in the compression zone of the section, while the corrugated steel mainly carries the tensile strain in the tension zone, allowing the material properties to be utilized more efficiently. A comparison between Figure 7a,b shows that, compared with concrete-only specimen C, the maximum compressive strain of the concrete in the CSC structure is smaller at the same load level. This indicates that the rigid support provided by the corrugated steel effectively shares the load borne by the concrete. A comparison between Figure 7c,d shows that, compared with corrugated steel-only specimen CS, the maximum tensile strain of the corrugated steel in the CSC structure is lower at the same load level. This confirms that the lateral confinement provided by the concrete to the corrugated steel effectively retards strain growth and delays the onset of yielding of the corrugated steel.

As shown in Figure 7b,d, when the structure reaches its ultimate bearing capacity, the strain distribution at the mid-span section of the CSC specimen remains continuous from the concrete in the compression zone to the corrugated steel in the tension zone. This indicates that the two materials maintain good synergistic action before failure of the CSC. After the CSC reaches the ultimate load, concrete cracking and interfacial delamination between concrete and corrugated steel occur. These phenomena are important causes of the abrupt change in strain distribution. A comparison of the maximum strain values of the three structures at ultimate load shows that the maximum strain of the CSC composite structure is significantly lower than those of the two single-material structures. This indicates that the composite action between corrugated steel and concrete effectively optimizes the stress–strain distribution across the section. It fully utilizes the mechanical advantages of the two materials and improves the overall bearing capacity and deformation resistance of the structure.

It is worth noting that in the experimental scheme of this study, only one specimen was used for each structural type. Therefore, the variability among specimens and the repeatability of the tests could not be evaluated. This imposes certain limitations on the experiment, and the test results obtained are responses under specific conditions. The value of the experimental scheme lies mainly in revealing mechanisms and generating hypotheses, rather than in providing overall performance or design conclusions.

## 4. Finite Element Analysis

### 4.1. Modelling

**Figure 8:**
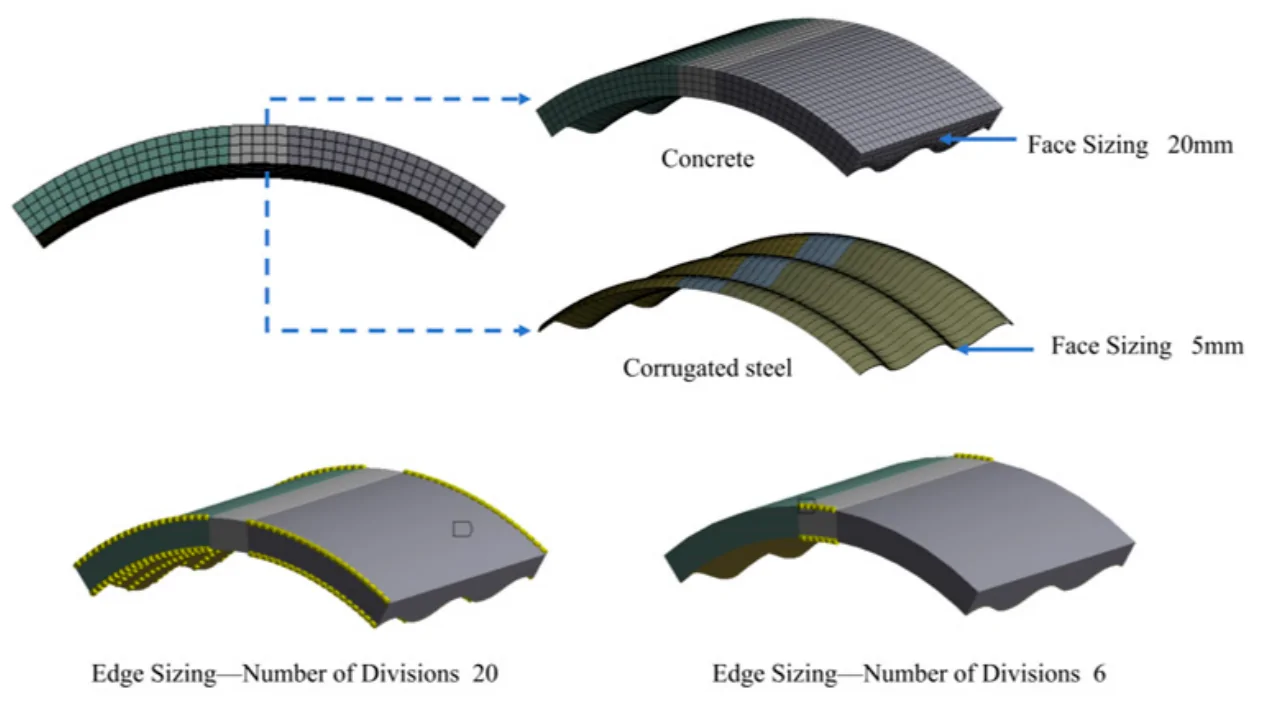
Mesh generation of finite element model.

The finite element analysis was performed in ANSYS Workbench 19.0. A three-dimensional numerical model was established according to the actual dimensions of the test specimens. In the numerical model, the concrete structure was located in the upper part and the corrugated steel structure in the lower part. The two components were modeled separately. After the geometry was completed, the concrete and corrugated steel were selected together and the Form New Part operation was performed. This merged the two bodies into a single Part, with shared topology and common nodes at the interface. Because the Form New Part approach with common nodes was used, no steel–concrete contact pair was generated in the model. Therefore, parameters such as contact formulation, normal contact stiffness, tangential stiffness, and friction coefficient were not defined. The interface load transfer is currently achieved directly through the shared nodes. The supports were simulated using remote displacements, and constraints were applied to the concrete and corrugated steel sections at both ends of the model. The element type used in the model was a solid element. To ensure computational accuracy, the mesh sizes were set as follows. The boundary lines at the mid-span loading position of the specimen were each divided into 6 divisions (as “Number of Divisions 6” in Figure 8). The boundary lines at the positions corresponding to the corrugated steel arc length were each divided into 20 divisions (as “Number of Divisions 20” in Figure 8). The section sizes of the concrete and corrugated steel at both ends of the specimen were set to 20 mm and 5 mm, respectively. The corrugated steel is divided into 109 mesh elements in the thickness direction, and the composite structure as a whole is divided into 16,125 mesh elements.

The nonlinear solution was performed using the Newton–Raphson method. Convergence was controlled by the L2 norms of the residual force and displacement increment, with tolerance coefficients of 0.9. The moment convergence check was deactivated. To accommodate severe local plastic deformation, the plastic strain increment limit was relaxed from the default 0.15 to 1 × 10^5^. This relaxed convergence control was used to improve numerical stability in the highly nonlinear local region, and the results were verified by mesh-sensitivity and load–displacement checks. For convergence control, the applied force and displacement were used to constrain the specimen deformation. When local deformation was large, the strain convergence criterion was relaxed.

The present model employs a shared-node perfect-bond assumption. Under this assumption, interfacial slip, debonding, and friction cannot be simulated. The associated conclusions are mainly intended for analyses of overall damage evolution trends and synergistic load-bearing mechanisms.

**Figure 9:**
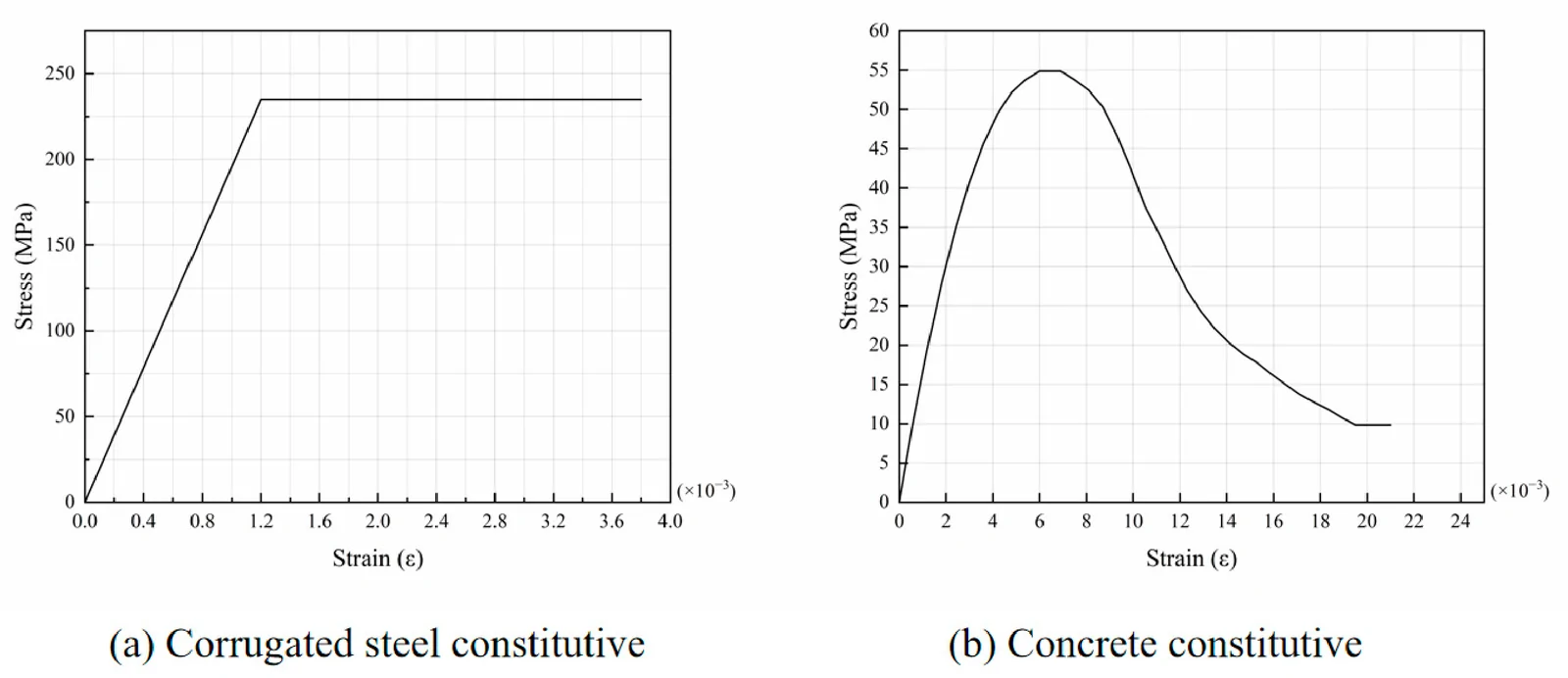
Constitutive model of (**a**) corrugated steel and (**b**) concrete.

Figure 9 illustrates the constitutive models adopted for the corrugated steel and concrete. Equations (1)–(3) define the compressive constitutive relations for concrete. For concrete in tension, a smeared crack model is employed, and crushing is disabled. No explicit damage variable or damage evolution equation is introduced in the model; the degradation of shear stiffness after cracking is instead considered indirectly through the crack shear transfer coefficients.

(1) $$\varepsilon_{i}=K_{2}\varepsilon_{i}^{*}$$

(2) $$\sigma_{i}=K_{1}\frac{f_{c}}{\sigma_{\mathrm{ref}}}\sigma_{i}^{*}$$

(3) $$K_{2}=\frac{\sigma_{i}^{*}K_{1}\left(f_{c}/\sigma_{\mathrm{ref}}\right)}{\varepsilon_{1}^{*}E}$$

In. *ε_i_* denotes the i-th actual strain control point.

*σ_i_* denotes the i-th actual stress control point.

*K*_1_ is the stress multiplier.

*K*_2_ is the stress multiplier.

*σ_ref_* is the reference stress value.

*f_c_* is the concrete cube compressive strength.

*E* represents the elastic modulus of the concrete.

**Figure 10:**
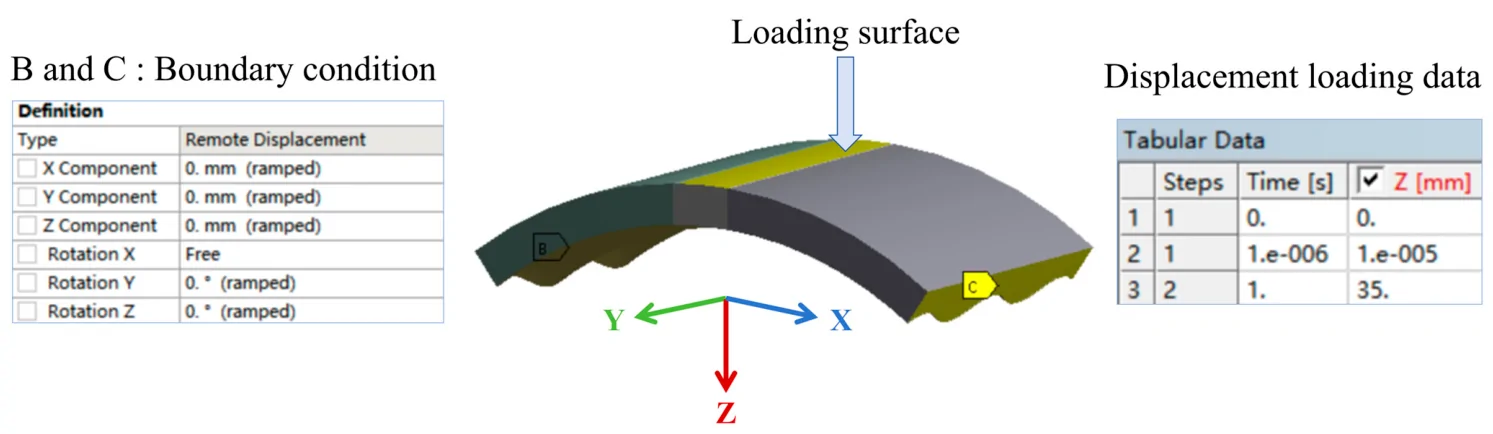
Loading method and boundary constraint diagram.

In the numerical analysis, the load was applied using the same displacement-controlled method as in the tests, with a displacement of 35 mm obtained from the tests applied to the loading surface. The boundary constraints were set according to the Boundary condition shown in Figure 10; that is, the displacements in the X, Y, and Z directions were constrained, and the degrees of freedom in the Y and Z directions were restrained. In the figure, the X direction is the span direction of the structure, the Y direction is the width direction of the structure, and the Z direction is the vertical direction.

### 4.2. Modeling Results

**Figure 11:**
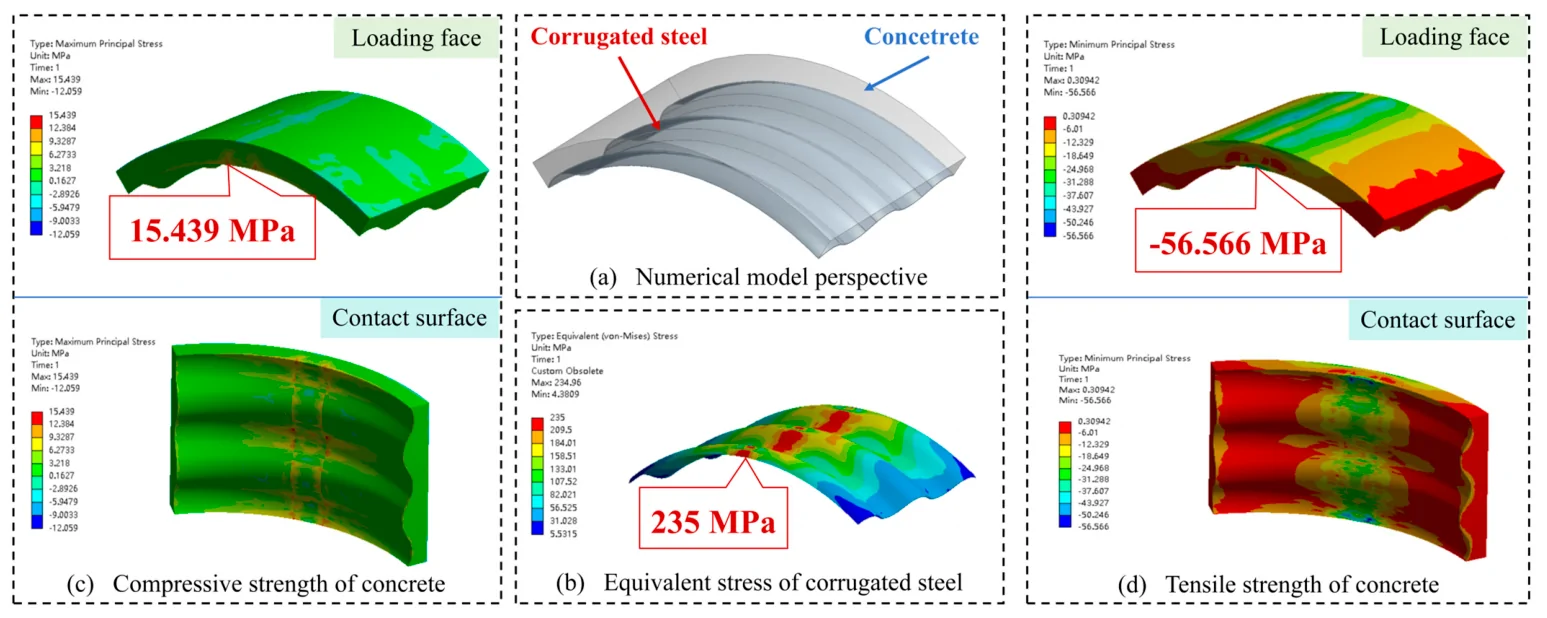
Structural stress contour. (**a**) Numerical model perspective and (**b**) equivalent stress of corrugated steel and (**c**) compressive strength of concrete and (**d**) tensile strength of concrete.

The structural stress contour obtained from the numerical analysis is shown in Figure 11. As can be seen from Figure 11a, the corrugated steel structure first reaches the yield strength of steel, 235 MPa, at the mid-span loading surface in contact with the concrete. As shown in Figure 11b,c, the compressive stress in the concrete at the loading surface and in the region in contact with the corrugated steel approaches the concrete compressive strength over a relatively large area. This is consistent with the phenomena observed in the composite structure, such as through cracks in the concrete at the loading surface and spalling at the interface with the corrugated steel.

**Figure 12:**
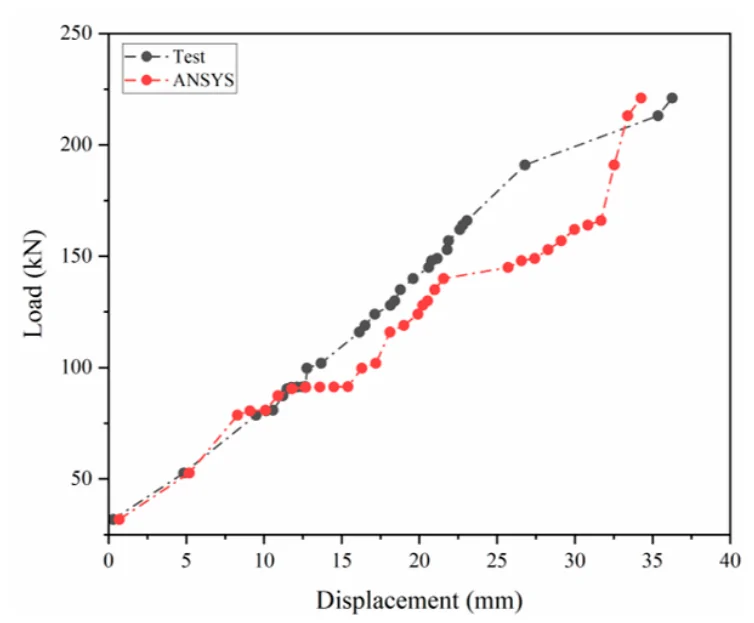
Comparison between the experimental results and the numerical analysis results.

The ultimate bearing capacities and mid-span load–displacement data obtained from the numerical simulation were further extracted and compared with the experimental measurements for validation. The results show that the simulated ultimate bearing capacity of the CSC specimen is 221 kN, with relative errors of no more than 2.3% compared with the experimental results. Figure 12 compares the load–displacement curves obtained from the tests and the numerical analysis. The curve obtained from the numerical analysis exhibits a variation trend basically consistent with that of the experimental curve. The development patterns at each stage and the mid-span displacement corresponding to the ultimate load follow the same trends as the experimental results. This indicates that the established three-dimensional numerical model can accurately reflect the actual mechanical characteristics and failure evolution process of the three types of arched structures, and can provide reliable support for subsequent mechanism analysis.

The numerical model established in Section 4.1 of this paper was developed solely for the geometric and loading conditions considered herein. It is applicable to the experimental study on the arched corrugated steel–concrete composite structures examined in this paper under mid-span concentrated loading. Within this research scope, the modeling method, boundary condition setup method, and load application method are all valid.

**Figure 13:**
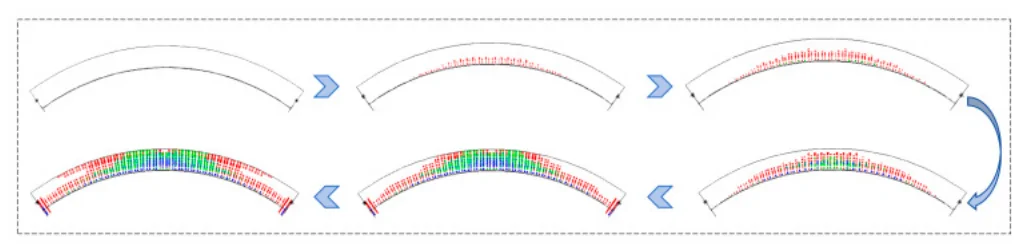
Structural damage development cloud map.

To further investigate the internal damage evolution of the composite structure throughout the loading process, damage distribution corresponding to different loads was extracted, as shown in Figure 13. In the initial damage stage, the stiffness difference between concrete and corrugated steel causes stress concentration near the interface. Concrete damage first reaches the damage threshold in this region. In the damage propagation stage, as the load increases, damage extends upward along the concrete height, and the damaged region continues to expand. In the failure stage, when the load approaches the ultimate bearing capacity, damage extends to the concrete surface and induces concrete cracking. In the tests, interfacial delamination between the corrugated steel and concrete was also observed in the CSC specimen. However, the numerical model in this paper adopts a shared-node perfect-bond assumption and does not explicitly simulate interfacial debonding. The above numerical damage evolution trend generally agrees with the experimentally observed failure morphology. This indicates that the two materials exhibit synergistic load-bearing characteristics and can provide a reference for the optimal design of composite structures. This numerical model uses the Solid65 concrete element to simulate the cracking behavior of the material. This element can track the cracking status of up to 3 mutually perpendicular planes at the integration points of the element. Under the action of load, when the principal tensile stress of a certain plane exceeds the tensile strength of the concrete, that plane will develop cracks; subsequent loading will cause the other orthogonal planes to also crack successively. The colors (red, green, blue) do not represent the crack width or damage degree, but are used to distinguish the planes where the cracks occur and the sequence of crack occurrence. Red represents the first group of cracks that appear first, usually the main tensile cracks; green and blue represent secondary cracks that occur later on other orthogonal planes, indicating that the element enters a complex stress state of multi-directional cracking. The arrows in the figure represent the progressive loading sequence, reflecting the gradual failure process of the arch structure from no cracks, to the occurrence of single-directional cracks locally, to the appearance of cracks in a large area.

### 4.3. Equivalent Discussion

To simplify structural modeling and numerical simulation analysis, three structural equivalence schemes are adopted in this paper to analyze differences in the results. All three schemes equivalently convert the original structure into an arched flat-plate structure. The equivalent structure is assigned concrete material properties, corrugated steel material properties, and homogeneous new material properties, respectively. The calculation process for the equivalent structural parameters is as follows.

When the structure is equivalently modeled with concrete material properties, the structural parameters are calculated as shown in Equations (4) and (5).

*E_C_I* = *E_CS_I_CS_* + *E_C_I_C_* (4)

*E_C_A* = *E_CS_A_CS_* + *E_C_A_C_* (5)

When the structure is equivalently modeled with corrugated steel material properties, the structural parameters are calculated as shown in Equations (6) and (7).

*E_CS_I* = *E_CS_I_CS_* + *E_C_I_C_* (6)

*E_CS_A = E_CS_A_CS_* + *E_C_A_C_* (7)

When the structure is equivalently modeled with the properties of the new material, the structural parameters are calculated as shown in Equations (8)–(10).

*EI* = *E_CS_I_CS_* + *E_C_I_C_* (8)

*EA* = *E_CS_A_CS_* + *E_C_A_C_* (9)

(10) $$\rho_{new}=\frac{\sum_{1}^{i}mi}{V_{new}}=\frac{\rho_{CS}V_{CS}{\;+\;\rho }_{C}V_{C}}{V_{CS}\;+\;V_{C}}$$

In, *E_CS_*, *E_C_* and *E* denote the elastic moduli of the corrugated steel, concrete, and overall structure, respectively.

*I_CS_*, *I_C_* and *I* denote the moments of inertia of the corrugated steel, concrete, and overall structure, respectively.

*A_CS_*, *A_C_* and *A* denote the cross-sectional areas of the corrugated steel, concrete, and overall structure, respectively.

*ρ*_CS_, *ρ_C_* and *ρ_new_* denote the mass densities of the corrugated steel, concrete, and new material, respectively.

*V_CS_* and *V_C_* denote the volumes of the corrugated steel and concrete, respectively.

**Figure 14:**
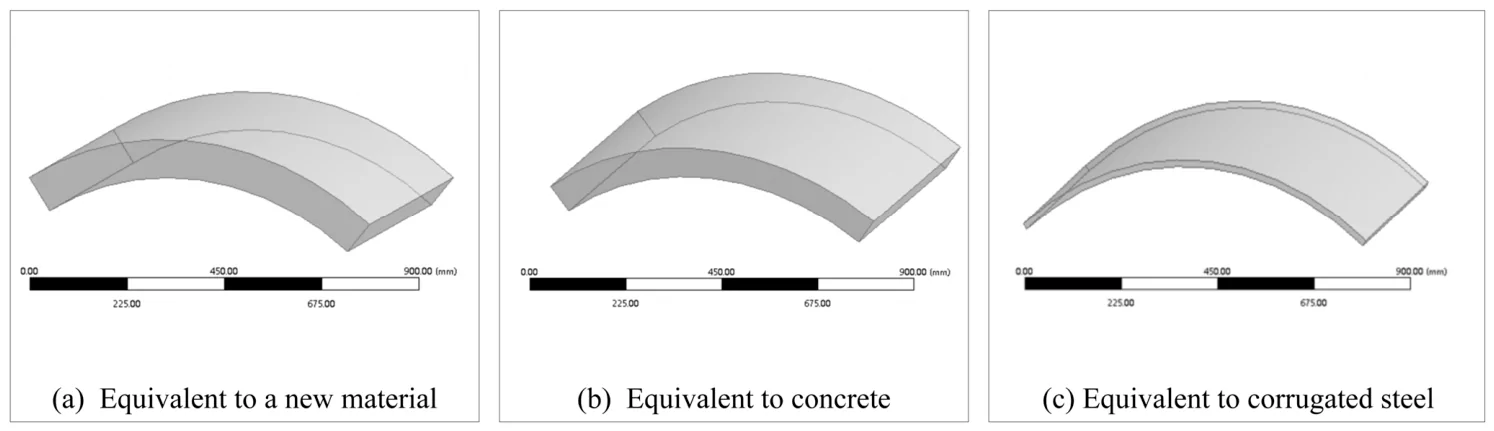
Three equivalent structural models. (**a**) equivalent to a new material, (**b**) equivalent to concrete, and (**c**) equivalent to corrugated steel.

The equivalent structural parameters were obtained for each scheme through calculation, and the corresponding numerical models were established, as shown in Figure 14.

**Figure 15:**
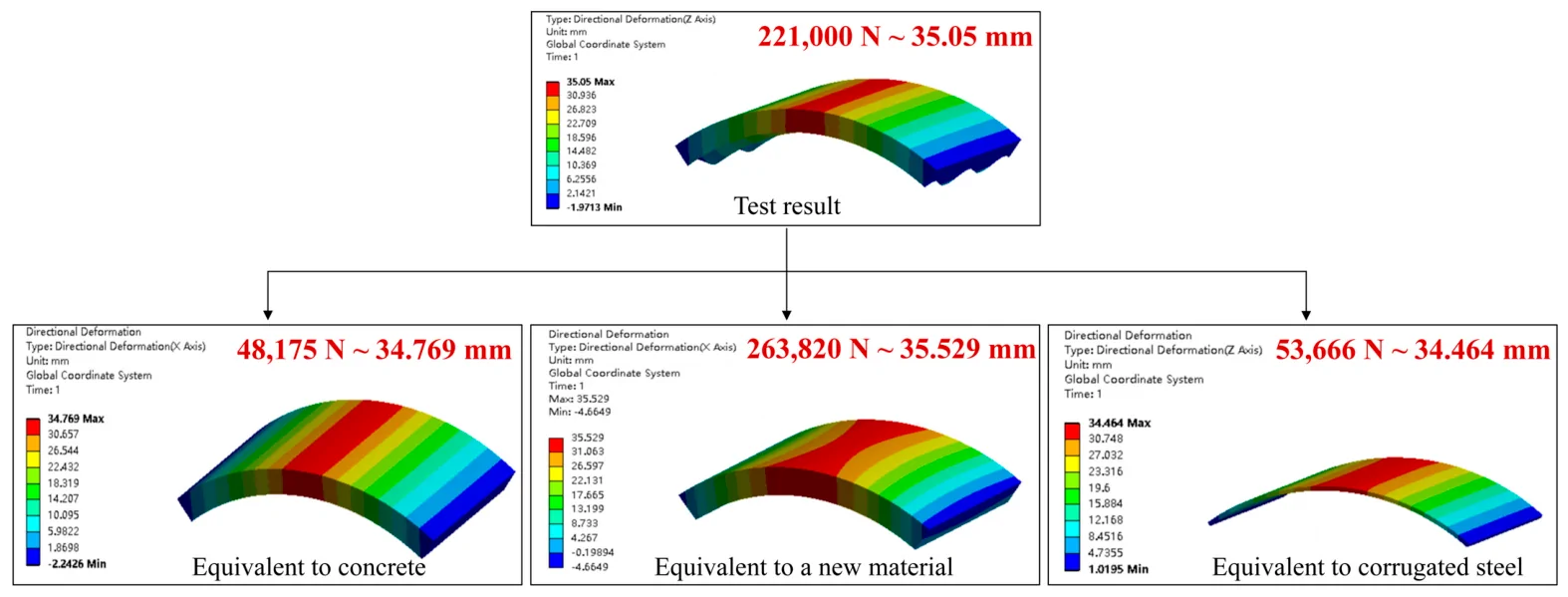
Three kinds of equivalent structure numerical cloud diagram.

The same loading conditions and boundary constraints as those in Section 4.1 were applied to each of the three equivalent structures. The resulting load–displacement responses are shown in Figure 15.

**Figure 16:**
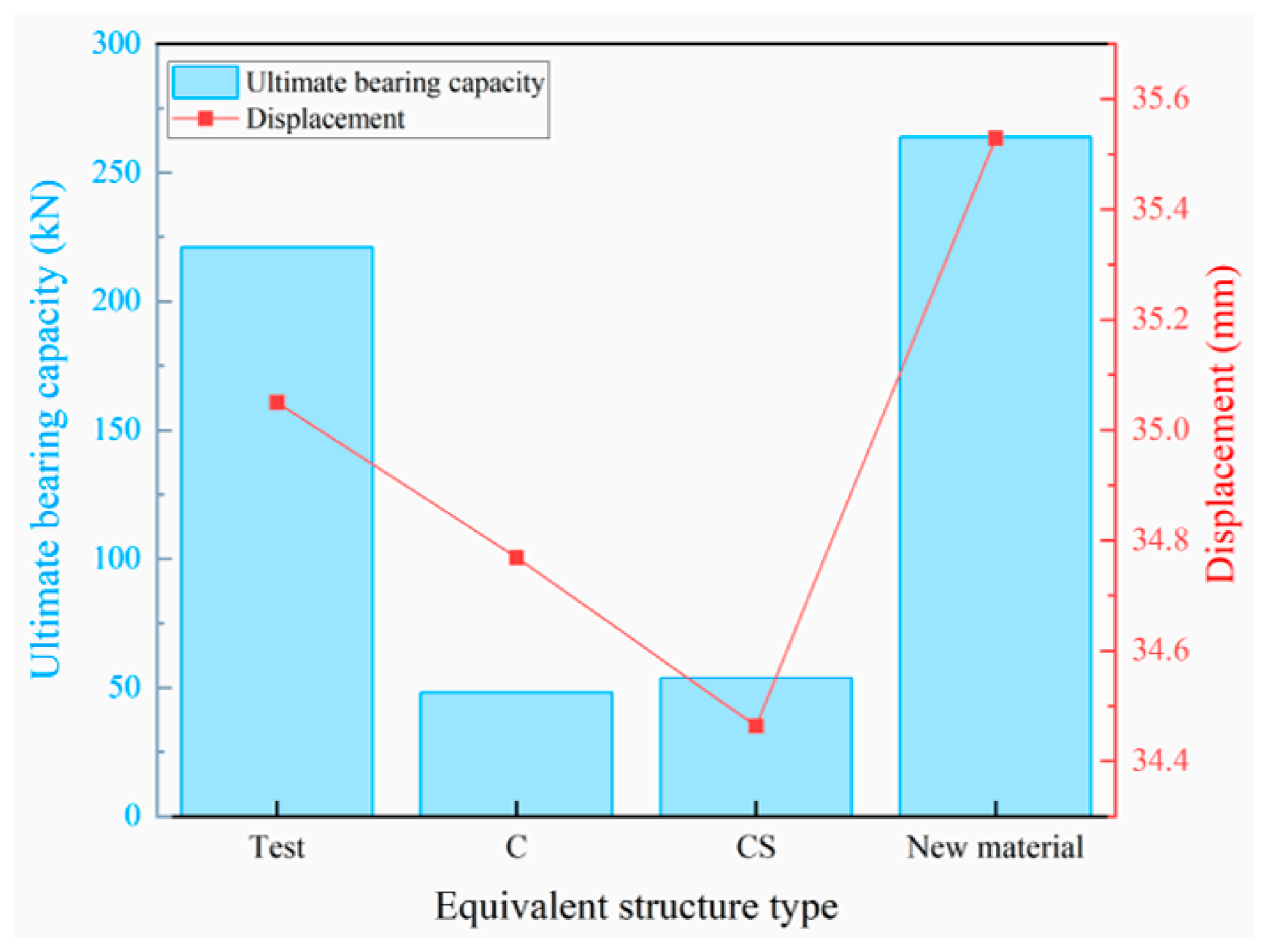
Comparison of load–displacement of three equivalent structures.

The simulated results of the three equivalent structures and the test specimen were summarized, and the resulting load–displacement comparison is shown in Figure 16. The results show that after the original structure was equivalently modeled as a concrete structure and a corrugated steel structure, the ultimate bearing capacities of the specimens decreased by 172,825 N and 167,334 N, respectively, compared with the original test specimen, and the maximum displacements decreased by 0.81% and 1.70%, respectively. In contrast, after the original structure was equivalently modeled as a new material structure, the ultimate bearing capacity increased by 16% and the maximum displacement increased by 1.35% compared with the original test specimen. Based on the data deviation characteristics, when the composite structure is equivalently modeled with new material properties, the deviations in ultimate bearing capacity and maximum displacement from the original structure are both within 20%. Although the computational accuracy is limited, the calculation results can be used for mechanism analysis and for judging structural failure trends.

In summary, when performing numerical analysis of the mechanical behavior of complex corrugated steel–concrete composite structures, the composite structure can be equivalently modeled as a homogeneous new composite material structure. The relevant sectional and mechanical parameters can be obtained through theoretical calculation. This equivalent method reduces the difficulty of numerical modeling of complex composite structures, decreases the workload of mesh generation and solution computation, and effectively improves analysis efficiency while ensuring computational accuracy. It can provide a simple and feasible simplified calculation method for the engineering design and numerical analysis of similar corrugated steel–concrete composite structures.

## 5. Conclusions

In this paper, loading tests were conducted on an arched corrugated steel–concrete composite structure, a concrete-only structure, and a corrugated steel-only structure. Combined with three-dimensional numerical simulation analysis, the load-bearing performance, deformation characteristics, strain distribution patterns, and failure mechanisms of the three types of structures were systematically investigated. The synergistic effect, displacement ductility, damage evolution, and equivalent analysis of the corrugated steel–concrete composite structure were emphatically studied. The following main conclusions are drawn:

(1) The corrugated steel–concrete composite structure exhibits higher load-bearing performance and a more stable synergistic load-bearing mechanism. The test results show that the ultimate bearing capacity of CSC is 216 kN, which is 517% and 52% higher than those of C and CS, respectively. In other words, compared with single-material members, the corrugated steel–concrete composite structure shows significantly enhanced overall flexural stiffness and ductility. The ultimate bearing capacity of the composite structure is not only higher than those of the concrete-only member and the corrugated steel-only member, but also higher than the algebraic sum obtained by linear superposition of their ultimate bearing capacities, demonstrating a clear positive synergistic effect. This fully indicates that this composite configuration offers significant combined advantages in stiffness, ductility, and bearing capacity.

(2) The established three-dimensional numerical model can characterize the actual mechanical behavior and failure evolution law of the corrugated steel–concrete composite structure. The relative error between the simulated ultimate bearing capacity and the experimental measurement is less than 3%, and the development trend of the load–displacement curves shows good agreement with the test results. The damage evolution process reproduced by numerical simulation presents the full process of the composite structure, from initial interface damage initiation and crack propagation and extension to final overall failure. Its evolution law is basically consistent with the failure modes observed in the tests, which can provide reliable support for mechanism analysis of similar structures.

(3) An equivalent simplified method for arched corrugated steel–concrete composite structures was proposed. The original composite structure was equivalently modeled as a homogeneous new composite material structure, and the relevant sectional mechanical parameters can be obtained through theoretical calculation. Compared with the original test specimen, the ultimate bearing capacity of the equivalent structure increased by 16%, and the maximum displacement increased by 1.35%. Although the computational accuracy is limited, the calculation results can be used for load mechanism analysis and for judging structural failure trends.

## Figures and Tables

**Table 1:** Mass percentage of concrete material components.

Material Categories	Fly Ash	Portland Cement	Silica Fume	Admixture	Rice Stone	Sand
Mass Percent	5%	30%	1%	0.8%	10%	53.2%

## Data Availability

The original contributions presented in this study are included in the article. Further inquiries can be directed to the corresponding author.
